# Intra-aortic balloon pump for ST-elevation myocardial infarction necessitating urgent coronary artery bypass grafting, still a valid indication?

**DOI:** 10.1007/s12471-024-01883-7

**Published:** 2024-07-02

**Authors:** Marie H. E. J. van Wijngaarden, Wim J. R. Rietdijk, Corstiaan A. den Uil

**Affiliations:** 1 Department of Cardiology, Maasstad Hospital, Rotterdam, The Netherlands; 2 Department of Hospital Pharmacy, Erasmus Medical Centre, Rotterdam, The Netherlands; 3 Department of Institutional Affairs, Vrije Universiteit, Amsterdam, The Netherlands; 4 Department of Intensive Care Medicine, Maasstad Hospital, Rotterdam, The Netherlands; 5 Department of Intensive Care Medicine, Erasmus Medical Centre, Rotterdam, The Netherlands

The intra-aortic balloon pump (IABP) has been used for decades as a low-risk, inexpensive and widely available cardiac mechanical support device for different indications. The primary aim in the acute coronary syndrome (ACS) population has been to improve myocardial perfusion through augmentation of diastolic flow [1, 2]. Several large-scale randomised controlled trials (RCTs) have been performed in patients undergoing elective high-risk percutaneous coronary intervention (PCI) and those with ACS with or without shock [3]. Most studies have failed to show a clinical benefit in patients on IABP support. In particular, IABP use has declined following publication of the results of the IABP-SHOCK II trial in 2012. Dutch single-centre pilot studies did, however, point towards benefit in patients with ACS and persistent ischaemia and those with non-ischaemic cardiogenic shock [2, 4, 5].

In this issue of the *Netherlands Heart Journal*, Hemradj and colleagues from the Isala Hospital in Zwolle, the Netherlands describe the results of their retrospective cohort study on the use of preoperative IABP (pIABP) in patients with ST-elevation myocardial infarction (STEMI) undergoing urgent (≤ 48 h following hospital admission) coronary artery bypass grafting (CABG) in the period 2000–2018 [6]. Multiple single-centre RCTs have explored the efficacy of pIABP in patients undergoing high-risk cardiac surgery. These studies focused on patients with impaired ejection fraction, unstable angina, a high EuroSCORE or significant left main stem stenosis, but not on STEMI. Hemradj et al. did include patients with STEMI with or without shock, [6] which is unique in the literature. In total, 69.5% of the patients (171/246; mean: 9 times p. a.) received pIABP, and 21.1% of the pIABP patients (36/171; mean: 6 times p. a.) received one after 2012. pIABP patients had a lower blood pressure, lower left ventricular ejection fraction, higher prevalence of thrombolysis in myocardial infarction (TIMI) flow post-PCI grade < 3 and higher EuroSCORE. Crude 30-day all-cause mortality was 12.6%, and there was no statistically significant difference between the pIABP and non-pIABP groups (*p* = 0.82). Using inverse probability treatment weighting (IPTW) to adjust for imbalance in covariates, the authors found a mortality benefit. with a relative risk of 0.52 (0.30–0.88) for 30-day mortality associated with pIABP use, which persisted at 1 year.

The authors do not present a flowchart with the number of all STEMI patients ending up with urgent CABG in the investigated time frame. This raises the question how frequently urgent CABG is performed in STEMI patients in the modern era. From the data presented by the authors, we can deduce that after 2012, a declining annual number of ~12 patients with STEMI underwent urgent CABG. The reasons why PCI had failed in these patients are not fully clear, but 50.6% of the pIABP patients had a TIMI flow post-PCI grade < 3.

From a methodological perspective, there are four questions that arise. In general, the Methods section could have been more elaborate, and that makes us wonder how to interpret the results. *First*, how did the authors assess normality of the continuous variables? This is fundamental as many advanced statistical models and tests depend on the normality assumption. Further, it is likely, but not fully clear from the tables, that continuous variables are reported as median (interquartile range). *Second*, the authors describe the imputation of missing data. They correctly claim that missing data may lead to several biases and loss of precision. It is not clear which variables were used in this step for the imputation. Therefore, the reader cannot know how, and under what assumptions, the imputation was performed and cannot value the imputation step. The latter may be assessed by the so-called fraction of missing information. This measure examines ‘the loss of information due to missingness, while accounting for the amount of information retained by other variables within a data set’ [7]. *Third*, the authors describe that they used linear regression—which is normally used for continuous outcome variables—to estimate associations with the binary outcome variable (i.e. 30-day mortality). It is not clear why the authors used this statistical model to estimate the effects and how a relative risk is actually derived from linear regression. It is known that binary logistic regression is most suitable to assess mortality. *Fourth*, the authors used IPTW to attempt to mitigate the effects of confounding. One may ask what the validity of the IPTW procedure applied to a relatively small sample size is. Of course, a study population of 246 patients is larger than the previously proposed cutoff number of 150 [8], but the number of covariates used by Hemradj et al. for the IPTW procedure is higher [8]. As the inclusion of more covariates requires a larger sample size, 246 may be too little to successfully apply such a procedure.

In summary, the study conducted by Hemradj and colleagues suggests that STEMI patients in whom PCI failed and who underwent urgent CABG may benefit from IABP. Ideally, this should be investigated in a large RCT. However, considering the decreasing number of STEMI patients requiring urgent CABG, this may not be feasible.
